# Synthetic computed tomography data allows for accurate absorbed dose calculations in a magnetic resonance imaging only workflow for head and neck radiotherapy

**DOI:** 10.1016/j.phro.2020.12.007

**Published:** 2021-01-11

**Authors:** Emilia Palmér, Anna Karlsson, Fredrik Nordström, Karin Petruson, Carl Siversson, Maria Ljungberg, Maja Sohlin

**Affiliations:** a Department of Radiation Physics, Institute of Clinical Sciences, Sahlgrenska Academy, University of Gothenburg, Gothenburg, Sweden; b Department of Medical Physics and Biomedical Engineering, Sahlgrenska University Hospital, Gothenburg, Sweden; c Department of Oncology and Radiotherapy, Institute of Clinical Sciences, Sahlgrenska Academy, University of Gothenburg, Gothenburg, Sweden; d Department of Medical Physics, Lund University, Malmö, Sweden; e Spectronic Medical AB, Helsingborg, Sweden

**Keywords:** Synthetic CT, Pseudo CT, MRI only, Head and neck, Treatment planning, Convolutional neural network

## Abstract

**Background and purpose:**

Few studies on magnetic resonance imaging (MRI) only head and neck radiation treatment planning exist, and none using a generally available software. The aim of this study was to evaluate the accuracy of absorbed dose for head and neck synthetic computed tomography data (sCT) generated by a commercial convolutional neural network-based algorithm.

**Materials and methods:**

For 44 head and neck cancer patients, sCT were generated and the geometry was validated against computed tomography data (CT). The clinical CT based treatment plan was transferred to the sCT and recalculated without re-optimization, and differences in relative absorbed dose were determined for dose-volume-histogram (DVH) parameters and the 3D volume.

**Results:**

For overall body, the results of the geometric validation were (Mean ± 1sd): Mean error −5 ± 10 HU, mean absolute error 67 ± 14 HU, Dice similarity coefficient 0.98 ± 0.05, and Hausdorff distance difference 4.2 ± 1.7 mm. Water equivalent depth difference for region Th1-C7, mid mandible and mid nose were −0.3 ± 3.4, 1.1 ± 2.0 and 0.7 ± 3.8 mm respectively. The maximum mean deviation in absorbed dose for all DVH parameters was 0.30% (0.12 Gy). The absorbed doses were considered equivalent (p-value < 0.001) and the mean 3D gamma passing rate was 99.4 (range: 95.7–99.9%).

**Conclusions:**

The convolutional neural network-based algorithm generates sCT which allows for accurate absorbed dose calculations for MRI-only head and neck radiation treatment planning. The sCT allows for statistically equivalent absorbed dose calculations compared to CT based radiotherapy.

## Highlights

• The geometry of the synthetic CT is comparable to the CT in the H&N region.
• Synthetic CT in the H&N region provides similar absorbed dose calculation as the CT.
• Absorbed dose calculations in the dental region could benefit from using synthetic CT.

## 1 Introduction

For a couple of decades, computed tomography data (CT) has been a cornerstone for external beam radiation treatment planning (RTP) as it directly provides Hounsfield Unit (HU) information that are converted to relative electron densities (RED) in the treatment planning system (TPS) and used for absorbed dose calculation. In CT of the head and neck (H&N) region, streak artifacts arising from dental fillings are common. Magnetic resonance imaging data (MRI) however are less prone to these types of artefacts and have superior soft tissue contrast compared to CT. MRI contribute with more consistent estimation of tumour volumes [1], [2], [3], but with the drawback of lower geometric fidelity.

Combined use of CT and MRI is beneficial in RTP but requires a registration between the data sets. These registrations may introduce errors [4], [5], [6], e.g. due to imperfect registration algorithms and differences in patient setup and geometry between the imaging occasions, which will systematically affect the entire treatment course.

Lately, interest has been directed towards MRI-only RTP; a workflow that involves MRI as the only imaging modality. The benefits of MRI-only RTP are a simplified faster workflow, and a decrease of the patientś radiation exposure. It might also be more cost effective [7], [8]. Development of MRI-only techniques would further be beneficial for MRI-linear accelerator treatment [7], [9], [10].

Since most TPSs require CT (i.e. HU) for absorbed dose calculations, a conversion from MRI to synthetic CT data (sCT) has been proposed using a variety of different methods [7], [8], [9]. The results are promising for several anatomical sites, e.g. prostate, brain, thorax and H&N [11], [12], [13], [14], [15], [16], [17], [18], [19]. However, many previous strategies for sCT generation have drawbacks: Bulk density assignment conversion requires for the vast majority previously published methods manual steps in order to separate the MRI data into different tissue classes, creating sCT with non-continuous HU [9]. Atlas-based conversion has high dependency on accurate registration and an inability to handle abnormal anatomy [9], while many of the voxel-based conversions use multiple and non-standard MRI acquisition sequences to generate sCT, which increases the MRI examination time [9]. To overcome such problems, convolutional neural network (CNN)-based methods to generate sCT have been proposed for brain, pelvis and H&N [20], [21], [22], [23], [24], [25]. CNN-based methods typically use a single standard MRI sequence, generate sCT fast, and can learn characteristics of the anatomy and its correlation with individual HU variations and therefore characterize abnormal anatomy in the sCT.

Previously published studies of sCT generation has focused mainly on pelvis and brain, and only a small number of studies on H&N [16], [24]. Furthermore, most previous work uses in-house developed software [19], [20], [21], [22], [23], [24] that are not clinically approved and not generally accessible. An available and trustworthy software for conversion of MRI data to sCT data would increase the clinical accessibility of an MRI-only workflow.

The transfer function estimation (TFE) algorithm [26] is a CNN-based method that allows for conversion from MRI to sCT. The aim of this study was to evaluate H&N sCT generated by the commercial TFE algorithm by validation of the geometry of the sCT, and the accuracy of the sCT data based absorbed dose calculations.

## 2 Materials and methods

### 2.1 Patient cohort

During 2018 and 2019, H&N cancer patients at our clinic were asked about study participation during their ordinary radiation therapy MRI simulation, resulting in 44 enrolled study participants (Table 1). Ethical approval was obtained from the Swedish Ethical Review Board (Reg nr. 645-17). Inclusion criteria were: Volumetric modulated arc therapy (VMAT) treatment plans, field of view (FOV) for both MRI and CT covering the patient́s body contour in the region of the H&N and shoulders, and no major post-surgical implants or dental restorations causing disruption of the body contour in the MRI.

**Table 1:** Patients characteristics and prescribed dose.

Characteristic		Patients	
		No	%
Sex	Male	28	64
	Female	16	36
Age, years	Mean	67	
	Range	39–85	
Cancer site	Oral	10	23
	Oropharynx	12	27
	Hypopharynx/Larynx	9	20
	Epipharynx	1	2
	Parotic gland	7	16
	Outer ear canal/Skin	3	7
	Unknown primary head and neck	2	5
Prescribed dose to primary tumour	68 (2 Gy/fx) Gy	3	7
	54 (3 Gy/fx) Gy	2	5
	46 (2 Gy/fx) Gy	1	2
Prescribed dose to primary tumour and lymph nodes	68 Gy + 68 (2 Gy/fx)	11	25
	68 Gy + 52.7 (1.55 Gy/fx) Gy	27	61

### 2.2 Image acquisition

CT images were acquired using an Aquilion LB CT scanner (Toshiba Medical Systems, Tokyo, Japan). MR images were acquired with a 1.5 T Aera wide bore MR-system (Siemens Healthcare, Erlangen, Germany), equipped with a flat tabletop (CIVCO Radiotherapy, Iowa, USA). Two flexible receiver coils were placed under (4 channels) and over (16 channels) the H&N and shoulder region. A coil bridge held the upper coil to avoid deformation of the patient body contour. As recommended by the sCT vendor, a T1 weighted Dixon Vibe (3D spoiled GRE) acquisition was used for sCT generation. For CT and MRI scan parameters, see Supplementary material.

The CT and MRI were acquired consecutively, and in treatment position, with the patient́s head and shoulders immobilized in a custom-made thermoplastic mask (Orfit Industries, Wijnegem, Belgium) and a head support (CIVCO Radiotherapy, Iowa, USA).

### 2.3 sCT generation

The commercial TFE algorithm used in this work estimates a spatially variant transfer function based on MRI, using a high-resolution three-dimensional deep CNN. The TFE algorithm applies the generated transfer function to the MRI, creating a CT representation. The TFE algorithm is previously described by the manufacturer [26], and is characterized by its ability to propagate high resolution details from the MRI to the generated sCT. In this work, the TFE algorithm was made available through a pre-release of MRI Planner version 2.2 (Spectronic Medical AB, Helsingborg, Sweden), which allows the generation of sCT to be integrated in the clinical workflow.

The TFE algorithm was trained using a multicenter database of 80 paired MRI and CT datasets, having comparable image contrast as used in this study. Significant data augmentation was employed during training to ensure that the trained network is robust to a large variety of MRI scanners. Augmentation included both conventional procedures such as scaling, deformation, and rotation as well as extensive proprietary procedures to mimic scanner and protocol specific properties and artifacts.

A part of the training data set was obtained within a pre-study of this project, with no overlap between training data and data set used in the present study. The processing time for sCT generation was approximately four minutes using an online cloud service, not including the time required to transfer the images to and from the cloud service. Processing was performed on servers equipped with Nvidia Tesla V100 GPUs with 16 GB RAM.

### 2.4 Image evaluation

For each patient, image evaluation was conducted for the planning target volume (PTV) with an additional 15 mm margin in craniocaudal direction covering significant parts of the high-dose region for coplanar 6 MV beams. Differences in patient positioning and anatomical appearance due to physiology (e.g. swallowing) between the MRI and CT imaging session was handled by rigid registration of the CT to the MRI followed by a multi-pass non-rigid registration (VelocityAI, Varian Medical Systems, Palo Alto, CA). The deformed CT data (CT_def_) was resampled to the reconstruction matrix and in-plane resolution of the sCT. The accuracy of the registration was assessed using crossfading and visual inspection. Body contours were obtained using the auto-segmentation tool in Eclipse TPS (Varian Medical Systems, Palo Alto, CA). Since the sCT is generated based on a generic HU-RED curve, CT_def_ was converted to achieve the same HU-RED relationship as for the sCT. In the CT_def_ and sCT, thresholds were set to extract bone (>250 HU), soft tissue (-200HU – 250HU), and air segments (<-200 HU). Evaluation was omitted for slices containing intense streak artefacts in the CT_def_.

The geometry of the generated sCT was evaluated (Matlab 2017b, Mathworks, Natick, MA, USA) with CT_def_ as reference. Mean error (ME), mean absolute error (MAE), Dice similarity coefficient (DSC) and Hausdorff distance (HD) were compared for overall body, soft tissue, bone, and air segments for CT_def_ and sCT. ME and MAE were calculated with segments obtained from the CT_def_, and the DSC and HD difference were calculated with both segments’ sets. Water equivalent depth (WED) was calculated for 2639 directions in 360° around the patient longitudinal axis. For every direction, the intensity profile from patient center to body contour was weighted with RED, using the CT data calibration curves for treatment planning, and summarized to WED. For each data set, the WED difference was calculated for three slices located at different anatomical regions: Vertebra Th1-C7, mid mandible, and mid nose. These three regions have different anatomical appearance and hence present various challenges for sCT generation and are clinically relevant since they comprise PTV locations for all patients included in this study.

### 2.5 Absorbed dose evaluation

To evaluate sCT as basis for absorbed dose calculation, difference in absorbed dose distribution for the original CT and sCT was calculated. For each patient, target volumes and organs at risk (OARs) were delineated on the CT using MRI-guidance, following clinical guidelines [27]. A 6 MV VMAT treatment plan was generated in Eclipse TPS with the original CT as basis (Table 1), using the anisotropic analytical algorithm (AAA 13.6.23) with a 2.5 × 2.5 mm grid for absorbed dose calculation. The calibration curve for the H&N CT protocol was used for CT data HU conversion to RED. All clinical treatment plans were approved according to local department practice. The clinical treatment plan and the delineated structures were transferred to the sCT using rigid registration in the TPS between the MRI and original CT. In order to minimize effects of different patient positioning between the MRI and CT imaging sessions, and achieve an impartial comparison, the body contour in the sCT was modified to resemble that of the CT by assigning soft tissue expanding outside the modified body contour to air and assigning air inside and connected to the modified body contour to water in the sCT. Recalculation of the absorbed dose was conducted for the sCT using identical beam parameters as the original CT based treatment plan. The calibration curve obtained from MRI Planner was used for sCT HU conversion to RED.

The CT and sCT data based absorbed dose distributions were compared by calculation of the relative local absorbed dose difference for a subset of dose volume histogram (DVH) parameters (Table 2) used in the Swedish Phase III multicenter randomized trial ARTSCAN III [27]. PTV_T_ (PTV for primary tumour), D_mean_ and D_2%_ for body were additionally evaluated. In agreement with the ARTSCAN III protocol, the PTV was not evaluated closer than 4 mm to the skin surface. OARs were evaluated only if receiving at least a mean absorbed dose of 10% of the prescribed dose (corresponding to 6.8 Gy). The absorbed dose distributions of the sCT and CT were compared using 3D gamma evaluation [28] with a 10% absorbed dose cut off, and a passing criterion of 2% local dose difference/1 mm.

**Table 2:** Evaluated dose volume histogram (DVH) parameters, mean relative absorbed dose difference in percent and difference in absolute absorbed dose.

DVH parameter	Relative absorbed dose Mean ± 1sd (%)	Absolute absorbed dose Mean ± 1sd (Gy)	No. of included volumes
PTV_T_ D_mean_	−0.1 ± 0.2	−0.1 ± 0.1	44
PTV_T_ D_98%_	−0.0 ± 0.4	−0.0 ± 0.2	44
PTV_T_ D_2%_	0.0 ± 0.4	0.0 ± 0.3	44
PTV_N_ D_98%_	−0.1 ± 0.2	−0.1 ± 0.1	27*
PRV Spinal cord D_2%_	−0.2 ± 0.2	−0.1 ± 0.1	44
Parotid Sin D_mean_	−0.2 ± 0.5	−0.1 ± 0.1	36**
Parotid Dx D_mean_	−0.2 ± 0.4	−0.0 ± 0.1	38**
Larynx D_mean_	−0.1 ± 0.2	−0.0 ± 0.1	40**
Upper esophageal sphincter D_mean_	−0.1 ± 0.4	−0.1 ± 0.1	37**
Submandibular glans Sin D_mean_	−0.3 ± 0.4	−0.1 ± 0.1	32**
Submandibular glans Dx D_mean_	−0.2 ± 0.3	−0.1 ± 0.1	38**
Body D_2%_	−0.1 ± 0.3	−0.1 ± 0.2	44

Abbreviations: planning target volume primary tumour (PTV_T_), absorbed dose (D), planning target volume lymph nodes (PTV_N_), planning organ at risk volume (PRV)

* Dose delivered using simultaneous integrated boost

** Organ surgically removed pretreatment or excluded

To evaluate the equivalence of the dependent and normally distributed calculated DVH parameters between the two data sets, a paired equivalence test of two one-sided t-tests [29] was conducted, with a 95% confidence interval (CI) (package Statsmodels v0.10.0, Python v3.7), and with the null hypothesis that the mean sCT data based absorbed dose distributions differs from the CT data based absorbed dose distributions. The absorbed doses for the evaluated DVH parameters were normalized, and the equivalence interval was set to (−1%, 1%).

## 3 Results

### 3.1 Image evaluation

**Fig. 1:**
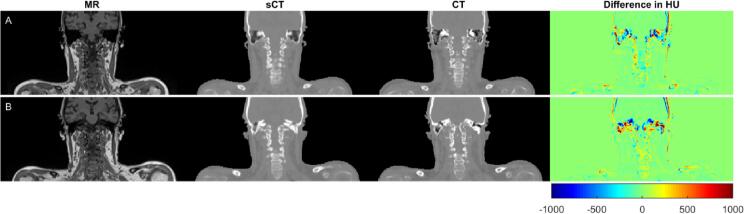
The magnetic resonance image (MRI), generated synthetic computed tomography (sCT) and computed tomography (CT) data, as well as corresponding Hounsfield units (HU) difference (sCT-CT), representing the A) maximum (102 HU) and B) minimum (47 HU) mean absolute error for overall body.

For all study patients meeting the inclusion criteria, sCT (Fig. 1) was successfully obtained. There were 12 patients excluded due to surgical implants or dental restorations that cause disruption of the body contour in MRI.

Evaluation of all data sets resulted in a mean ME (±1 standard deviation, sd) of −5 ± 10 HU for overall body, −1 ± 7 HU for soft tissue, −62 ± 28 HU for bones, and 107 ± 75 HU for air. The mean MAE was 67 ± 14 HU for overall body, 38 ± 6 HU for soft tissue, 195 ± 27 HU for bone, and 198 ± 68 HU for air. The maximum and minimum MAE for overall body was 102 HU and 47 HU, respectively (Fig. 1).

Mean DSC (±1sd) for all data sets was calculated to 0.98 ± 0.05 for overall body, 0.95 ± 0.05 for soft tissue, 0.80 ± 0.07 for bones, and 0.81 ± 0.1 for air. The mean HD (±1sd) difference found for all data sets was 4.2 ± 1.7 mm for overall body, 5.2 ± 1.4 mm for soft tissue, 4.6 ± 1.2 mm for bones, and 2.8 ± 0.8 mm for air.

**Fig. 2:**
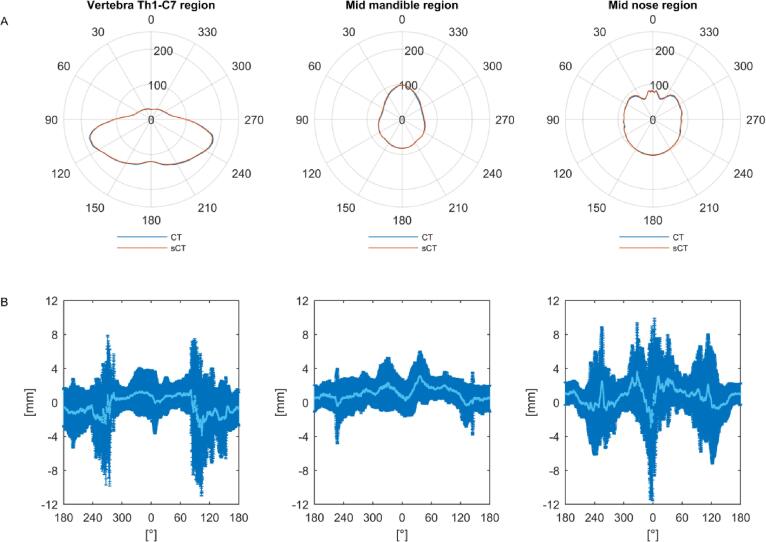
A) Mean water equivalent depths (WED) for all computed tomography (CT) data sets (blue) and corresponding synthetic CT (sCT) (red), and B) mean WED difference (light blue) and the corresponding standard deviation (sd) (dark blue) for 44 paired data sets as a function of degree, presented for three regions; Th1-C7 (left), mid mandible (middle) and mid nose (right). (For interpretation of the references to colour in this figure legend, the reader is referred to the web version of this article.)

The radial evaluation of WED in the vertebra Th1-C7 region showed a mean difference (±1sd) between sCT and CT_def_ of −0.3 ± 3.4 mm. For the mid mandible and mid nose regions, the corresponding mean difference was 1.1 ± 2.0 mm and 0.7 ± 3.8 mm. The difference in WED was largest in the right-left direction for the vertebra Th1-C7 and in the anterior-posterior direction for the mid nose region (Fig. 2).

**Fig. 3:**
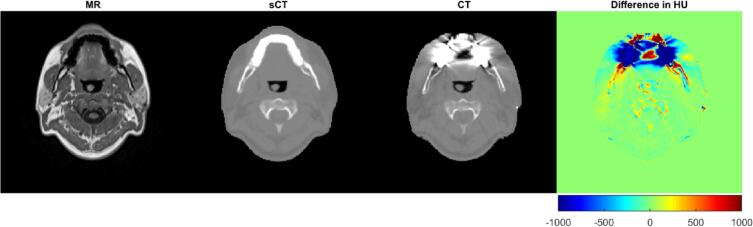
The magnetic resonance image (MRI), generated synthetic computed tomography (sCT) data with minor dental artefacts, computed tomography (CT) data with severe dental artifacts and difference between sCT and CT in Hounsfield units (HU).

In the sCT, signal void artefacts caused by dental fillings expand only a few millimeters, compared to the CT where streak artefacts typically impact the image quality several centimeters from the fillings (Fig. 3).

### 3.2 Absorbed dose evaluation

**Fig. 4:**
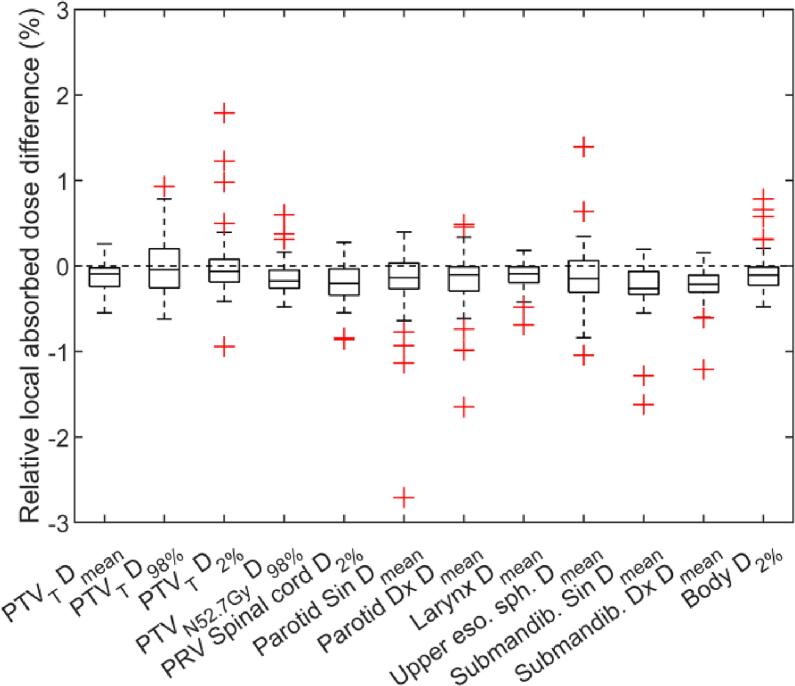
Relative absorbed dose difference for the evaluated dose volume histogram (DVH) parameters comparing synthetic computed tomography (sCT) and computed tomography (CT) dose distributions for 44 paired data sets. Abbreviation: planning target volume primary tumour (PTV_T_), absorbed dose (D), planning target volume lymph nodes (PTV_N_), planning organ at risk volume (PRV), Upper esophageal sphincter (Upper eso. sph), Submandibular glans (Submandib.)

Comparison of sCT and CT based treatment plans showed overall lower absorbed doses for the DVH parameters corresponding to the sCT based treatment plan (Table 2). The mean deviation in absorbed dose ranged from −0.3% (corresponding to −0.1 Gy) to 0.02% (corresponding to 0.01 Gy) for all DVH parameters. According to the paired statistical test of equivalence, both t-tests null hypothesizes was statistically rejected (p-value < 0.001). The data falls within the equivalence bonds (−1%, 1%) and is considered equivalent with differences close to zero compared to CT. The relative absorbed dose differences in percent for the selected DVH parameters, comparing sCT and CT dose distributions are shown in Fig. 4. There were 34 outliers (>1.5∙interquartile range) present for the parameters excluding PTV_T_ D_mean_. The 3D gamma evaluation (2%/1 mm criteria) gave a mean passing rate for sCT against CT of 99.4% (range: 95.7% to 99.9%).

## 4 Discussion

Since MR images lack electron density information required for treatment planning, a reliable CT substitute is required in order to enable transition from the traditional MR-CT based external radiation treatment workflow to an MRI-only workflow. In this study, we aimed to evaluate if synthetic CT data generated from MRI using a CNN-based TFE algorithm can be used for treatment planning of H&N cancer by evaluating geometric and absorbed dose differences between the sCT and CT. The geometric evaluation showed that the sCT are very similar to the CT, and the absorbed dose evaluation showed high agreement between dose distributions calculated using the different image sets.

An available and approved software for conversion of MR data to sCT data increases the clinical accessibility of an MRI-only workflow, compared to in-house developed software which have been used in most previously published studies of sCT generation [20], [21], [22], [23], [24], [25]. Previous work has mainly focused on pelvis and brain, and only a small number of studies on H&N [16], [25], possibly due to the combined challenges in patient positioning, large MRI FOV required and complex anatomy.

Dinkla et al. [25] generated sCT in the H&N region using a patch-based deep neural network where the architecture of the network was derived from a 3D U-Net, trained on 22 paired T2 weighted MRI and CT data sets. Via threefold cross validation, they obtained sCTs for 34 patients. Their overall body mean ME was 9 ± 11 HU, MAE was 75 ± 9 HU and mean DSC values was 0.98 ± 0.01, which agrees with the results obtained in this study. Farjam et al. [16] obtained similar overall body MAE, although using a multi-atlas-based approach to generate sCT in the H&N region.

Two main reasons for the difference in mean WED between the CT_def_ and the sCT (Fig. 2) have been identified: Firstly, uncertainties in the location of the body contour and other boundaries within the body (e.g. trachea) between MRI and CT_def_ have large impact on the results for short WED:s. This leads to relative differences in WED that is not related to the sCT generation. Secondly, difficulties in distinguishing bone and air in the MRI could lead to ambiguous sCT data representation of these voxels and this could explain why the ME showed a systematical under- and overestimation in HU of bone and air, respectively.

When evaluating the sCT geometry, the CT was non-rigidly registered to the MRI which is challenging in the H&N region [30]. For evaluation of absorbed dose differences, the sCT was instead rigidly registered to the CT in order to preserve the integrity of the original CT. Rigid registration cannot remediate simultaneous differences in positioning of chin and shoulder which was partly compensated for by using a corrected body contour in the sCT. Without this modification, delineated structures located closely to the skin surface, e.g. the PTVs, could end up outside the body contour. This would lead to a less impartial comparison between the sCT and CT data based absorbed dose distributions. There will, however, still be some remaining anatomical differences contributing to deviations in both geometric and absorbed dose evaluations not related to the quality of the sCT. Nevertheless, in an operating MRI-only workflow such registrations will be redundant.

For absorbed dose calculation in the H&N region, both atlas- and CNN-based methods for sCT generation have shown acceptable results [16], [25]. We have evaluated absorbed dose using DVH parameters as these results easily can be interpreted in the clinical setting. They are, however, not directly comparable to the voxel-wise approach used by Dinkla et al. [25] which, to the best of our knowledge, the only published article with an extensive absorbed dose evaluation of sCT generated with a CNN-based method in the H&N region.

By presenting the calculated absorbed dose result as the relative absorbed dose difference between the sCT and CT based treatment plans, the evaluation of absorbed dose to OARs becomes independent of the prescribed dose to target which allows for more general conclusions. However, large relative absorbed dose differences may have only minor impact in clinical practice, e.g. the left parotid D_mean_ outlier of −2.7% (Fig. 4) corresponded to a difference of 0.3 Gy. If the difference was evaluated relative to the prescribed absorbed dose, the result would have been −0.5%. All outliers for all parallel OARs had a mean absolute dose deviation below 0.4 Gy, which is low compared to the clinical constraints for these OARs.

All ten PTV outliers (Fig. 4) were evaluated in detail and the following outliers were identified to be caused by the sCT generation: For the 1.8% outlier, it could be concluded that the deviation arose since the CT possessed approximately four times higher HU in the front teeth dental filling than the sCT. The outlier of 1.23% was caused by having the trachea as part of the PTV and a difference in volume and position of the air in the trachea between CT and MRI acquisition. When replacing the air in the CT and sCT with water, the difference was decreased to −0.2%. For the outlier of 1%, D_2%_ was located in a dental filled tooth where CT possessed higher HU than the sCT (14518 versus 1334 HU). The outlier of −0.9% was caused by a spacer in the mouth, barely visible in the MRI and therefore not represented in the sCT, leading to a systematic shift of the treatment plan in the mouth region. To our knowledge the training data set did not include patients with a spacer at the time of our evaluation.

The commonly accepted total absorbed dose uncertainty in radiotherapy is 3–3.5% (1sd) [31], [32]. The maximum deviation in absorbed dose distributions for all DVH parameters evaluated in this study was 2.7%. The difference in absorbed dose for PTV related DVH parameters was within the previously suggested criterion on accepted dose difference of 2% for reliable use of MRI-only [33].

In the majority of our cases, sCT was accurately generated but some minor visual dissimilarities to CT were found. These differences might be an effect of limited training data for patients with abnormal anatomy or artificial features. The maximum difference in PTV_T_ D_mean_ for the dissimilar sCTs was 0.7% (0.5 Gy), which was considered to have minor impact in clinical practice.

For RTP based on CT, streak artifacts arising from dental fillings are usually handled by semi-automatic selection of the area and assignment of the artefacts to water density. In the sCT, artefacts were only present within a few mm in close proximity to the dental filling (Fig. 3). Therefore, patients with treatment sites in the dental region could benefit from using sCT based dose calculation as it contributes with more correct HU compared to CT. Potentially, there are other artifacts in the MRI data, such as motion artefact from breathing, swallowing, etc., however, this type of artifacts was not observed in this study.

In addition to the absorbed dose evaluation of the sCT performed in this study, it is necessary to characterize the possible MRI distortions, evaluate the impact of the workflow on the patient positioning on the treatment unit as well as setting up a quality assurance of the sCT when no CT is available [34], [35] before implementing a clinical MRI-only workflow.

In conclusion, our results show that the studied CNN-based TFE algorithm for generation of sCT can be used for dose calculations in an MRI-only workflow for H&N radiation therapy. The geometry of the sCT proved to be comparable to the CT in the H&N and shoulder region, and the absorbed dose evaluation confirmed that the sCT allow for accurate absorbed dose calculations and were statistically equivalent compared to CT.

Founding

This work was supported by research grants from Vinnova, Sweden’s innovation agency (2016-03847), through the national project Gentle Radiotherapy, the 10.13039/501100010223King Gustav V Jubilee Clinic Research Foundation, the Eurostars programme of the European Commission (E! 12326 ILLUMINUS) and the Swedish Childhood Cancer Found (MT2016-0015).

## Declaration of Competing Interest

The authors declare that they have no known competing financial interests or personal relationships that could have appeared to influence the work reported in this paper.
