# Supplementary material for: Synthetic computed tomography data allows for accurate absorbed dose calculations in a magnetic resonance imaging only workflow for head and neck radiotherapy
Source: Phys Imaging Radiat Oncol. 2021 Jan 11;17:36–42. doi: 10.1016/j.phro.2020.12.007 (PMC8058030; doi:10.1016/j.phro.2020.12.007)
Supplement: Supplementary Data 1 [file mmc1.pdf]

Appendix A Supplementary material

CT scan protocol parameters

CT parameter	Value
Reconstruction matrix	512x512 mm^2
Reconstruction in-plane resolution	0.90-1.2 mm^2
Number of slices	176-253
Slice thickness	2.0 mm
Slice spacing	0.0 mm
Peak kilo voltage output	120 kV
Exposure	225 mAs
Exposure time per slice	0.75 s

MRI scan protocol parameters

MRI parameter	Value
Sequence type	Spoiled GRE (VIBE)
Acquisition type	3D
Scan plane	Transversal
Frequency FOV	500 mm
Phase FOV	500 mm
Acquisition matrix	448x381
Reconstruction matrix	448x448
Reconstructed in-plane resolution	1.12x1.12 mm^2
Number of slices	198
Slice thickness	2.0 mm
Slice spacing	0.0 mm
Readout bandwidth	795 Hz/pixel
Number of echoes	2
Echo time	2.39 ms and 4.77 ms
Repetition time	8 ms
Number of averages	3
3D geometry correction	On
Total scan time	9 min 57 s
